# Chinese medicine for coronavirus disease 2019 as complementary therapy: A protocol for a systematic review and meta-analysis

**DOI:** 10.1097/MD.0000000000021034

**Published:** 2020-08-14

**Authors:** Min Zhou, Qijun Liang, QiuLan Pei, Fan Xu, Hang Wen

**Affiliations:** a College of Clinical Medical, Jiangxi University of Traditional Chinese Medicine; b Affiliated Hospital of Jiangxi University of Traditional Chinese Medicine, Nanchang; c College of Clinical Medical, Shanghai University of Traditional Chinese Medicine, Shanghai, China.

**Keywords:** Chinese medicine, coronavirus disease 2019, meta-analysis, protocol, systematic review

## Abstract

**Background:**

The aim of this systematic review and meta-analysis is to assess effectiveness and safety of Chinese medicine (CM) as complementary therapy in treating coronavirus disease 2019 (COVID-19).

**Methods:**

The following databases will be searched: PubMed, Cochrane, Embase, China National Knowledge Infrastructure, Chinese Science and Technology Periodical Database, and Wanfang database from October 1, 2019 to March 1, 2020. Randomized trials and quasi-randomized or prospective controlled clinical trials of CM that reported data on COVID-19 patients will be included. Study selection, data extraction, quality assessment, and assessment of risk bias will be performed by 2 reviewers independently. Odds ratios and correlative 95% confidence intervals will be calculated to present the association between the CM and CWM using Review Manager version 5.3 when there is sufficient available data.

**Results:**

The results will be disseminated through a peer-reviewed journal publication.

**Conclusion:**

This systematic review findings will summarize up-to-date evidence for that CM is more effective and safe as adjunctive treatment for patients with COVID-19.

**Ethics and dissemination:**

Ethics approval and patient consent are not required as this study is a systematic review based on published articles.

**PROSPERO registration number:**

CRD42020185382.

## 1 Introduction

Coronavirus disease 2019 (COVID-19), a novel and acute infectious disease, is currently producing an outbreak of pandemic proportions, whereby nearly 4.4 million people have already been infected worldwide until May 10, 2020.^[1–3]^ The pulmonary syndrome was subsequently named COVID-19 by the World Health Organization. The COVID-19 outbreak has been a serious threat to global public health. It has raised more attention on the epidemic and has caused widespread panic. Recent statistic analysis showed the mortality rate is 2.1% with a median survival time of 6.4 days.^[4–6]^ Since December 2019, it has led to a rising rate of pneumonia cases due to its characteristic of infectiousness. COVID-19-infected pneumonia is characterized by flu-like symptoms such as fever, cough, severe acute respiratory distress syndrome, and even death, most of them had a positive prognosis.^[7–9]^ Unfortunately, presymptomatic or asymptomatic transmission of severe acute respiratory syndrome coronavirus 2 (SARS-CoV-2) has been reported.^[10,11]^ Mass isolation, forceful measures, and advanced treatment play a vital in this event. After the outbreak of COVID-19, the state administration of traditional Chinese medicine (TCM) organized experts to make a TCM therapeutic scheme after careful consideration and discussion. Subsequently, some patients were cured after TCM treatment, which promoted the widespread application of TCM in patients with COVID-19 pneumonia. On January 27, 2020, the General Office of the National Health and Health Commission of China and the Office of the State Administration of TCM issued “Diagnosis and Treatment of Pneumonia Caused by Novel Coronavirus Infection.” This included a scientific and detailed TCM treatment plan and required local health committees to implement and strengthen the integration of TCM and CWM.^[12]^

Nevertheless, due to the “personalized” characteristic of TCM treatment, it is difficult to formulate standard treatment rules, resulting in uncertainty of TCM clinical efficacy. Therefore, it is necessary to conduct rigorous and objective quality evaluation for different types of clinical studies, and the results of analysis obtained on this basis are more convincing. With respect to previous review, the main outcomes were evaluated based on TCM symptoms, which were subjective without nucleic acid analysis results. In addition, there were more retrospective studies and fewer RCTS in previous studies. This review was conducted based on more RCTS with relatively more objective outcome assessment such as the disappearance time of main symptoms and nucleic acid. The review was updated according to the present evolution for providing a robust evidence base for clinical practice in treating COVID-19 pneumonia.

## 2 Methods

This systematic review was registered on PROSPERO (CRD42020185382) on May 11, 2020. The registered website for this protocol is https://www.crd.york.ac.uk/PROSPERO/.

### 2.1 Search strategy

Six databases including PubMed, Cochrane, Embase, China National Knowledge Infrastructure, Chinese Science and Technology Periodical Database, and Wanfang database were searched from October 1, 2019 to March 1, 2020. If any, we would try to contact the original study authors for the information we need. What is more, we would perform a manual search to track the references of relevant literature. Then we browsed the detail of the abstract and the full text, and selected eligible studies according to the inclusion criteria. The detailed search strategy for PubMed is demonstrated in Table 1. Similar search strategies would be built for other electronic databases.

**Table 1:**
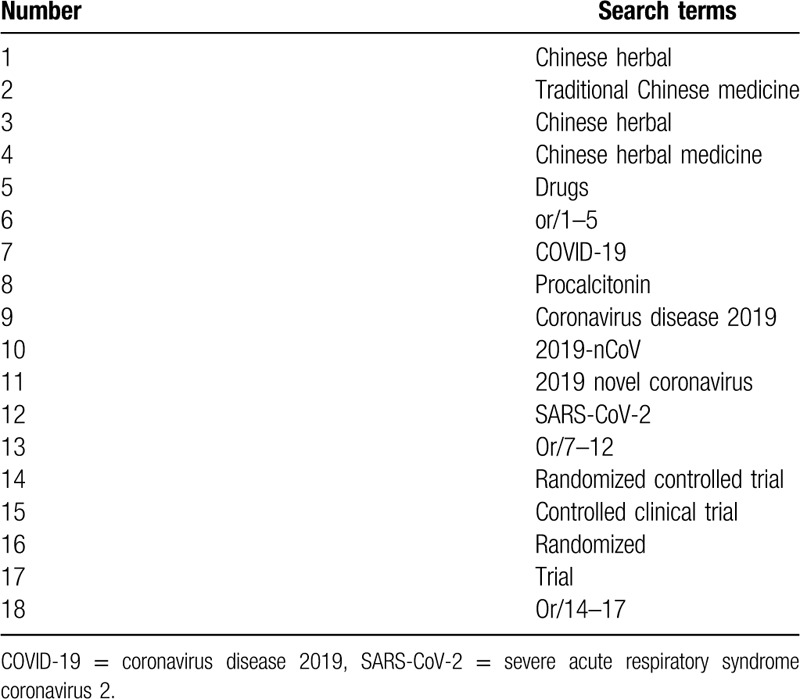
Search strategy for PubMed.

### 2.2 Including and excluding criteria

#### 2.2.1 Including criteria

1. Studies: Randomized trials and quasi-randomized or prospective controlled clinical trials that have tested traditional Chinese herbal medicine with or without western medicine for COVID-19 will be included. There will be no restrictions for blinding, follow-up, or publication status. Publications in English and Chinese will be included.
2. Participants: Patients diagnosed with COVID-19 referred to Diagnosis and Treatment of Pneumonia Caused by Novel Coronavirus Infection (Trial Version 5),^[13]^ without immediately life-threatening comorbidities will be included.
3. Interventions: Traditional Chinese herbal medicine involving a variety of forms, including pill, injection, capsule, and decoction. There will be no restrictions with respect to the type of comparator.
4. Outcomes: Our primary outcomes were effective rate, rate of severe illness, and adverse events. We will also assess the following secondary outcomes: days to disappearance of fever, days to disappearance of cough, days to be negative of nucleic acid, and length of stay in hospital. If other outcomes were reported in the eligible studies, these will be extracted and reported but we will give particular attention to the possibility of selective reporting bias when using any such outcomes in our review.^[14–21]^

#### 2.2.2 Exclusion criteria

1. Patients with life-threatening comorbidities likely to lead to death within the trial follow-up period
2. Duplicated data or data that cannot be extracted after contacting original authors
3. Case reports, reviews, mechanisms, unqualified interventions, and animal model experiments

### 2.3 Data abstraction

**Figure 1:**
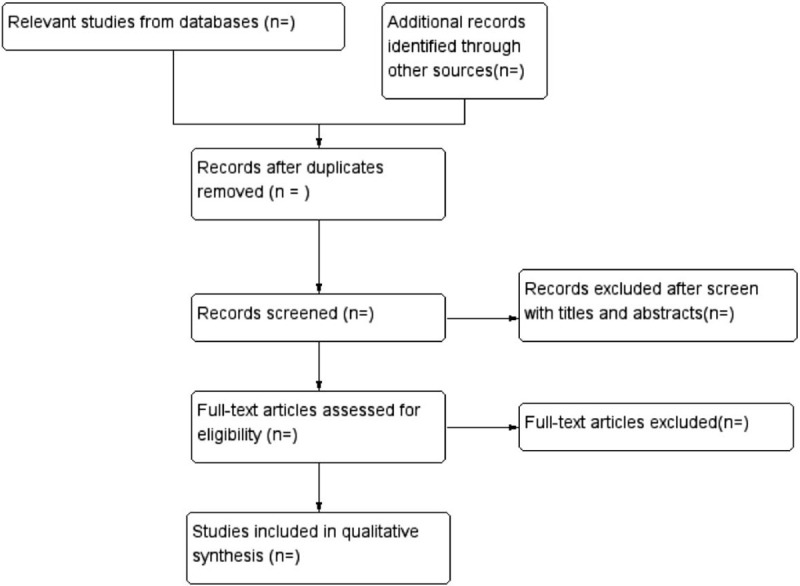
Flowchart of study selection.

Relevant information was extracted and cross checked by 2 independent reviewers (MZ and FX). The extracted data included the first author, sample size, age, interventions, outcomes, duration, randomization method, blinding of participants and personnel, allocation concealment, incomplete outcome data, follow-up, dropout and withdrawal, and adverse events. If there were disagreements between 2 reviewers, a 3rd reviewer (QL) was available to check for accuracy. Details of the selection procedure for studies were shown in a Preferred Reporting Item for Systematic review and Meta-analysis protocol (PRISMA-P) flow chart (Fig. 1).

### 2.4 Quality assessment

The assessment was performed by RevMan 5.3.3 according to the cochrane handbook. The overall assessment was based on details including random sequence generation, blinding of participants and personnel, allocation concealment, incomplete outcome data, selective reporting, and other biases. If we encountered differences, we would discuss with the 3rd reviewer (QL) and finally reach an agreement. The quality assessment was graded as “high” risk, “low” risk, or “unclear” risk depending on the degree of information provided.

### 2.5 Statistical analysis

Statistical analysis was conducted by the Review Manager 5.3 and stata 12.0 software. In this meta-analysis, the mean difference and the odds ratio were adopted to evaluate continuous variable outcomes and dichotomous outcomes with a 95% confidence interval. *P* < .05 was to be of statistical significance. There was no heterogeneity between studies (*I*^2^ < 50%), the fixed effect model was adopted; otherwise the random effect model was used. Considering that the treatment effect may be related to the treatment time, the data were analyzed by subgroup according to different intervening measure. Sensitivity analysis was conducted to evaluate the impact of the included studies on the final outcome. Forest plots and Egger test was conducted to assess potential publication bias. If *P* < .05, this was considered to be statistically significant.

### 2.6 Grading of Recommendations Assessment, Development and Evaluation quality assessment

The quality of evidence of outcomes will be assessed according to the Grading of Recommendations Assessment, Development and Evaluation system. The GRADE system includes 5 items: the risk of bias, inconsistency, indirectness, imprecision, and publication bias. The quality of evidence will be rated as “high,” “moderate,” “low,” or “very low.”

## 3 Discussion

Based on the basis of summarizing the experience of TCM in the treatment of SARS, the General Office of the National Health and Health Commission of China and the Office of the State Administration of TCM encouraged the combination of TCM and CWM.

This review was conducted based on the basis of the existing COVID-19 disease with an overview of the application of TCM for treating COVID-19 patients. In the treatment of COVID-19, TCM as an adjuvant therapy can significantly improve the effective rate, reduce the rate of severe disease, shorten the disappearance time of fever and cough, as well as the hospital stay of patients, and the most objective index is to significantly shorten the time of nucleic acid to be negative. In terms of adverse reactions, there is no significant difference with western medicine, indicating that TCM is safe as an adjuvant therapy. Nevertheless, the experimental group has released adverse reactions along with the increase of serum transaminase on 14 patients in study,^[2]^ and the control group released adverse reactions only on 8 patients. In this study, the experimental group is treated with Abby dole and Lianhua Qingwen Jiaonang, and the control group is treated by Lianhua Qingwen Jiaonang alone. Abby dole manuals shows drug incidence of adverse events is about 6.2%, mainly for nausea, diarrhea, dizziness, and elevated serum transaminase. In this study, no records of clinical adverse reactions were found in all cases except elevated serum transaminase, suggesting to some extent that the safety of the combined application of the 2 drugs is acceptable, but it also suggests that we need to do a good exploration in pharmaceutical care during the process of medication. On the whole, the data analysis shows that the adverse reactions mainly occur in the application of CWM, while the application of TCM is relatively safe and less adverse reactions occur.

Since the COVID-19 epidemic has not been completely subside and more large-sample clinical studies are still under way, the evidence of the current analysis has not yet been persuasive. After the epidemic is fully controlled and more research results are included in the analysis, we look forward to further updating and supplementing the systematic evaluation.

## Author contributions

**Conceptualization:** Qiulan Pei, Hang Wen.

**Data curation:** Qiulan Pei, Hang Wen.

**Formal analysis:** Min Zhou, Fan Xu.

**Funding acquisition:** Qijun Liang.

**Investigation:** Qiulan Pei.

**Methodology:** Min Zhou, Fan Xu.

**Software:** Qiulan Pei, Min Zhou, Fan Xu.

**Writing – original draft:** Min Zhou, Fan Xu.

**Writing – review & editing:** Min Zhou, Fan Xu, Qijun Liang.
